# Influence of Artificially Generated Interocular Blur Difference on Fusion Stability Under Vergence Stress

**DOI:** 10.16910/jemr.12.4.4

**Published:** 2019-09-11

**Authors:** Miroslav Dostalek, Karel Fliegel, Ladislav Dusek, Tomas Lukes, Jan Hejda, Michaela Duchackova, Jiri Hozman, Rudolf Autrata

**Affiliations:** Center of Paediatric Ophthalmology BINOCULAR, Litomysl; Czech Republic; Masaryk University, Faculty of Medicine, Dept. Optometry and Orthoptics, Brno, Czech Republic; Czech Technical University in Prague, Faculty of Biomedical Engineering, Dept. Biomedical Technology, Czech Republic; Czech Technical University in Prague, Faculty of Electrical Engineering, Dept. Radioelectronics, Czech Republic; Masaryk University, Faculty of Medicine, Institute of Biostatistics and Analyses, Brno, Czech Republic; École Polytechnique Fédérale de Lausanne; Switzerland; Masaryk University, Faculty of Medicine, Dept. Pediatric Ophthalmology, Brno, Czech Republic

**Keywords:** binocular fusion efficiency, vergence demand, blur balance, blur conflict, suppression, binocular rivalry, signal strength, natural image statistics

## Abstract

The stability of fusion was evaluated by its breakage when interocular blur differences were presented under vergence demand to healthy subjects. We presumed that these blur differences cause suppression of the more blurred image (interocular blur suppression, IOBS), disrupt binocular fusion and suppressed eye leaves its forced vergent position. During dichoptic presentation of static grayscale images of natural scenes, the luminance contrast (mode B) or higher-spatial frequency content (mode C) or luminance contrast plus higher-spatial frequency content (mode A) were stepwise reduced in the image presented to the non-dominant eye. We studied the effect of these types of blur on fusion stability at various levels of the vergence demand. During the divergence demand, the fusion was disrupted with approximately half blur than during convergence. Various modes of blur influenced fusion differently. The mode C (isolated reduction of higher-spatial frequency content) violated fusion under the lowest vergence demand significantly more than either isolated or combined reduction of luminance contrast (mode B and A). According to our results, the image´s details (i.e. higher-spatial frequency content) protects binocular fusion from disruption by the lowest vergence demand.

## Introduction

Binocular fusion is a key mechanism of normal vision. The proper combination of monocular signals is a crucial step preceding higher associative processes. Sufficient qualitative similarity of the images in the two eyes is pre-requisite of fusion [1]. One of the obstacles to fusion frequently seen in clinical practice is interocular differences in retinal images generated by uncorrected differences between refraction in the two eyes (anisometropia). The mismatching of images in two eyes can cause suppression and when sustained may cause amblyopia [2].

Since the original description by Wheatstone [3], much research effort has been directed at binocular rivalry: the alternating perceptual dominance generated by reciprocal suppression of conflicting signals from corresponding retinal points. The main focus in research is on the neural processes of suppression and awareness during stable retinal stimulation and their neuroanatomy [4]. The neuroimaging and electrophysiological studies concurwith psychophysical research. Whereas the early phases of spatial frequency and other first order signal processing are located to the central occipital region [5], second order and associative analysis progressively engages the lateral occipital cortex, temporo-parietal junction and higher association cortical areas [6]. This neuroanatomy supports a highly probable relationship of binocular rivalry to other suppressive phenomena such as motion-induced blindness [7] and dichoptic masking [8]. The latter is assumed to share with binocular rivalry the processing abilities of monocular neurons at V1 [9-11]. 

The same low level of main action is proposed for interocular blur suppression (IOBS) [12]. IOBS is a complex suppressive phenomenon described firstly by Abadi [13] and lately by Legge [14] as a "physiological process, where a stimulus of a given contrast presented to one eye can invariably prevent the detection of a lower image quality but otherwise identical stimulus presented to the other eye" [15]. They regarded IOBS as a variety of dichoptic masking [16]. 

Blur is characterized in terms of the spatial frequencies in an image as a particular pattern of selective loss of higher-spatial frequencies. Images of natural scenes have characteristic spatial frequency (f) spectra, with amplitude tending to fall off as 1/f. During rivalrous competition, natural images prevail over images with altered spatial statistics [17]. IOBS behaves in a similar way: monocular blur causes suppression of the image in that eye [12, 18]. 

A small degree of blur, related, for example, to physiological diplopia, is an intrinsic feature of retinal images [19], but blur can be much greater in some pathological conditions. Uncorrected refractive error and optical media opacification are the most frequent clinical situations where blur affects vision. Sufficiently large interocular blur differences in these cases may activate IOBS, which manifests as a disruption of binocular fusion. Artificially created targets or intentionally degraded natural images simulating blur can serve then for experimental or semi-clinical examinations of fusional characteristics [12, 20].

We have implemented an experimental design where artificially generated interocular blur difference generates measurable stress on binocular fusion. During dichoptic presentation of static images of natural scenes, progressive degradation of the image stimulating the non-dominant eye increases differential blur, step by step, until fusion is lost. We created three modes of ‘blur’ by manipulating the spatial and contrast structure of a natural grayscale image. Mode A simulates natural blur. In mode B contrast was reduced over the whole spatial frequency range of the test image, and in mode C only high spatial frequency content was reduced. In each of the three conditions, the interocular blur differences needed to disrupt fusion was measured under various levels of vergence demand in healthy observers. 

The current study was designed to assess our hypothesis that different modes of artificial interocular blur imbalances in an images‘ quality differs in their capacity to disrupt binocular fusion. The long-term aim of the study was to inspire novel ideas for optimization of antisuppresive approaches in binocular pleoptics where the conventional use of diffusive foils (clinically known as the Bangerter’s filters or partial occluders) is recently being converted into dichoptic training with virtual reality devices (VRD). Technologically advanced treatment is based on dichoptic balancing of the amblyopic eye’s blur (caused by the anisometropic retinal image defocus and consequent cortical deterioration related to IOBS) with the artificially degraded simulation image delivered by haploscopic display to the non-amblyopic eye. The clinical standard is to use the stimulation image artificially blurred equally to the natural degradation generated by uncorrected refraction error [21]. There is an ample body of sound evidence that amblyopic eye has a complex defect of the contrast sensitivity combining the reduction of contrast sensitivity and the decrease in high-spatial frequency content in the perceived image. The same changes in contrast sensitivity generates Bangerter’s filters [22] and our mode A. Due to nearly unlimited potency of VRD in the image quality modulation, the clinical question is how to further strengthen dichoptic training by stimulation image modulation. The aim of our study is to compare suppressive potency of the isolated reduction of contrast (our experimental mode B) and high-spatial frequency content (our experimental mode C) in comparison with the clinical standard (our mode A).

## Methods

### Ethical approval

The Local Independent Ethics Committee of the Litomysl Hospital, Litomysl, Czech Republic, approved project Vliv umělé degradace fixačního obrazu a relativní vergenční zátěže na práh dichoptického maskování [Influence of the Fixation Image’s Artificial Degradation and the Relative Vergence Demand to Dichoptic Masking‘s Threshold]. It approved the experimental protocol, information form, and informed consent form for adult subjects and parents or guardians of teenage subjects, and attested their conformity to the Declaration of Helsinki.

### Equipment

**Figure 1:**
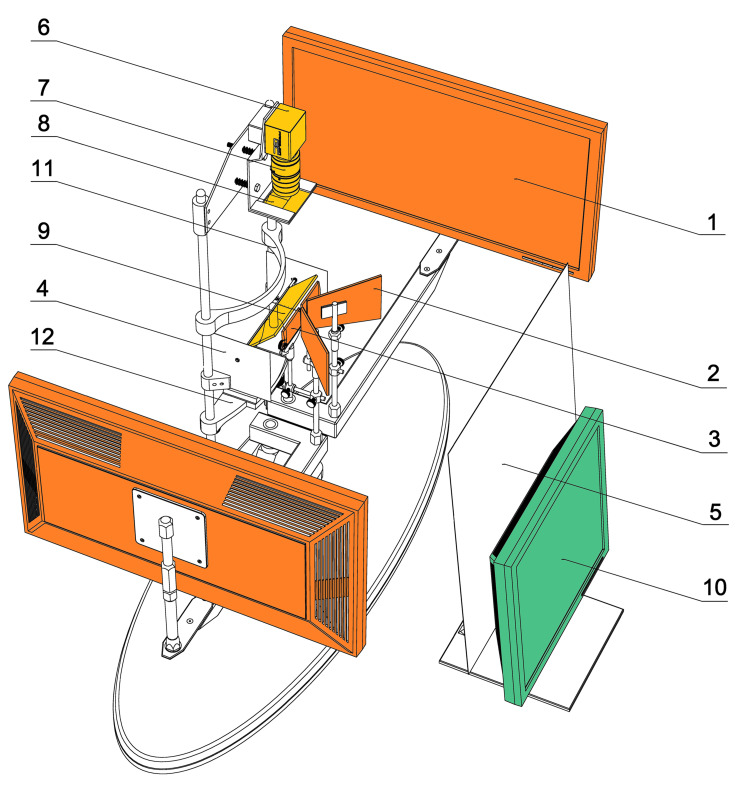
Custom-made experimental PC-haploscope. (1) stimulation monitor, (2) first surface mirror, (3) convex lens, (4) lateral shield, (5) front shield, (6) monochromatic CMOS camera, (7) manual zoom and focus lens, (8) eccentric photo refraction shield, (9) dichroic mirror, (10) controlling monitor, (11) headrest, (12) chinrest.

All measurements were performed with a custom-made PC-haploscope [23] (Fig 1). The stimulation part of this experimental device consists of two identical 22“ LCD monitors SyncMaster E2220N (Samsung Electronics Co., Ltd., Suwon, South Korea) operating in Full-HD (1920 x 1080 px, luminance 300 cd/m^2^, contrast 1000:1, dynamic contrast 70000:1), custom-made trapezoidal first surface planar mirrors, and + 2.0 D biconvex lenses mounted on two arms independently rotating about axes passing through the centres of rotation of each eye. The real distance of monitors (0.45 m) from subject´s eyes was optically converted by the lenses into the virtual viewing distance (2.0 m) located between the resting point of accommodation (RPA) and optical infinity for comfortable observation of LCD monitors. A Prosilica GC750 CMOS video camera (Allied Vision Technologies GmbH, Stadtroda, Germany) with recording rate of 67 frames per second (752x480 px), manual zoom and focus lens Pentax C6Z1218 (Ricoh Co., Ltd., Tokyo, Japan), custom-made eccentric photo refraction shield equipped with four NIR LED 530E850C (Hebei I.T. Co., Ltd., Shanghai, China) with maximal irradiation at 850 nm and a dichroic (“hot”) mirror (Edmund Optics Inc., Barrington, NJ, USA was used for continuous monitoring of the eye position. The head of each subject was stabilized with a headrest during the experiment. The control unit of the PC-haploscope is based on standard PC (OS Windows XP, Microsoft Co., Redmond, WA, USA) and custom-made software using Microsoft.NET Framework (Microsoft Co., Redmond, WA, USA). 

### Stimuli

**Figure 2:**
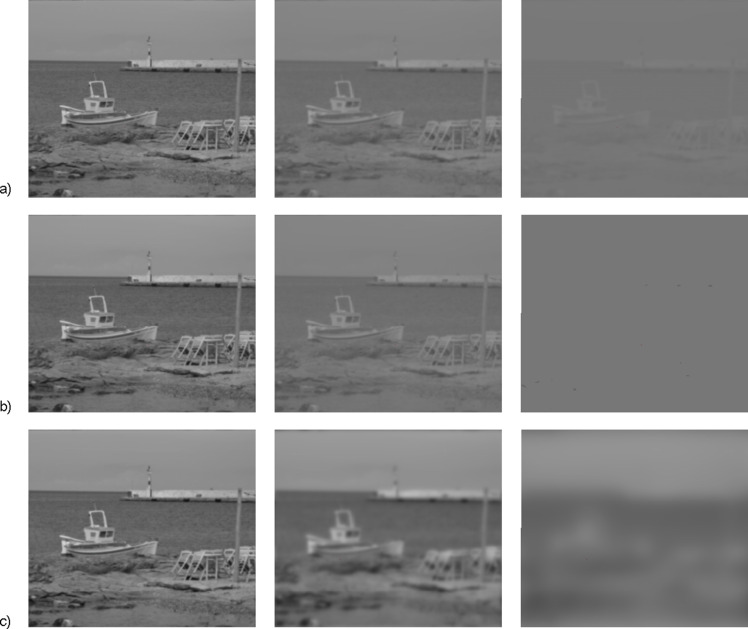
Modes of progressively degraded series of the stimulating images.The progression of experimental images’ degradation is illustrated by third (1.00 FCT), eleventh (3.67 FCT) and twentieth (6.67 FCT) images from the sequences of twenty four images. a) reduction of both contrast and high spatial frequency content (mode A) b) isolated reduction of contrast (mode B) c) isolated reduction of high spatial frequencies (mode C)

For determining the degree of interocular blur needed to disrupt fusion (the “IOBS threshold”) we used a series of twenty-four progressively more degraded images. We artificially created three modes of visual stimulation differing in reduced clues for binocular fusion: parallel reduction of contrast and high spatial frequencies in mode A, isolated lowering of contrast in mode B and isolated decrease of high spatial frequencies in mode C (Fig 2). The angular sub tense of the experimental images was 70 (w) x 62 (h) degrees (1145 x 1022 px). 

**Figure 3:**
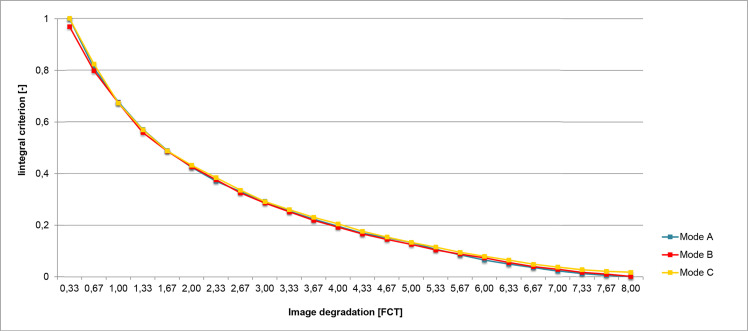
Objective comparison of test image degradation in three test modes by integral criterion. Mode A: parallel reduction of contrast and high spatial frequencies, mode B: isolated reduction of contrast, mode C: isolated reduction of high spatial frequencies.

The artificial degradation of the stimulating images was attained by the setup of frequency responses of the transformation filters based on the human contrast sensitivity function (CSF). The spatial frequency spectrum of the original image was calculated using a two-dimensional discrete Fourier transform (2D DFT). To form the degraded images, the spectral coefficients of the 2D DFT were weighted by the amplitude frequency response of the image transformation filters [24]. Changing the filter parameters allowed us to obtain three series of differently degraded images. To assure objective matching across test series, the integral criterion was employed as an objective measure of test image degradation independent of degradation mode. (The integral criterion was calculated as the area under the amplitude frequency response of the transformation filter within the considered range of spatial frequencies. The area under the investigated amplitude frequency response, i.e., for the selected degradation mode and degradation level, is then normalized by the area under the curve for the lowest degradation level.) The filter parameters were set-up to obtain the integral criterion’s progression parallel for mode B and mode C to reference mode A (see Fig 3). 

In the absence of generally accepted metrics for a quantitative description of the subjective perception of blur, we have proposed FCT unit for our experiments. The sample stepwise progression of an image’s degradation is obtained by the progressive multi-layering of diffusive foils. We refer to the subjective blurring effect of a single diffusive foil as 1 FCT, each grade thereafter represents one added foil and their summed diffusive effect. This principle is protected by Industrial Property Office of the Czech Republic [25]. The principle is used clinically for fusion cover test (FCT) and the unit adopted it’s acronym [26]

Our concept of matching the objective blur’s measure expressed by integral criterion to subjective metrics in CSF units is based on subjective matching of the artificially blurred stimulating image of reference mode A and empirically generated blur by interposing of multi-layered diffusive foils between the eye and the non-blurred image (performed by four ophthalmologically health subjects, co-authors MD, KF, JH and TL). Every stimulating image obtained individual designation related to its degradation degree expressed in integral criterion as well as in FCT units. The physiological, exponential relation between subjectively perceived blur expressed in units FCT and objective metric system based on integral criterion is illustrated in Fig 3.

### Procedure

**Figure 4:**
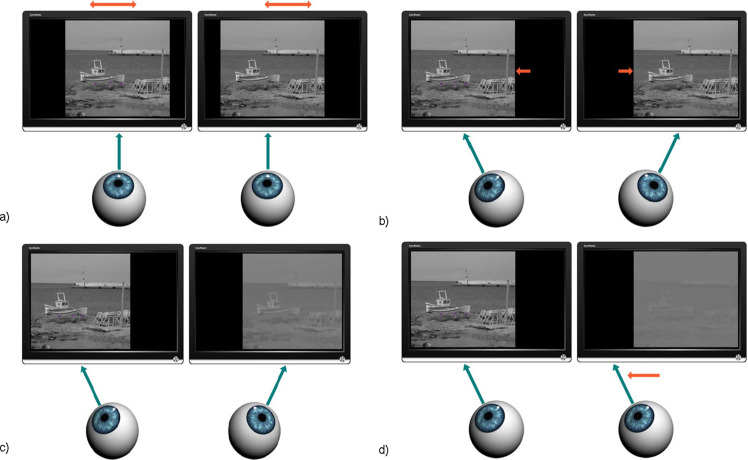
Experimental design of dichoptic suppressive threshold detection. a) compensation of subject’s phoria and accommodation convergence b) set-up of vergence demand c) progressive degradation of non-dominant eye’s image d) suppression and leaving forced vergence position of non-dominant eye

The measurement procedure started with neutralization of subject´s phoria and small accommodation convergence associated with the virtual viewing distance. Placing a dissociative prism (4 Δ base down) in front of the dominant eye induced transient fusional disruption and vertical diplopia. Any horizontal motoric imbalance was manifested by relative horizontal image dislocation. The haploscope arms were rotated to fully compensate any horizontal component of induced diplopia (Fig 4A).

**Figure 5:**
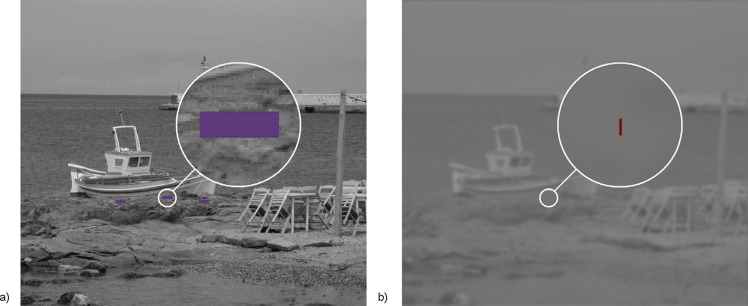
Experimental images presented simultaneously in two stimulation monitors. a) dominant eye (non-degraded image with violet box) b) non-dominant eye (degraded image with red line)

Prior to each measurement of the dichoptic suppressive threshold, the vergence demand level was randomly selected from the alternatives 4, 8, 12, 16 Δ of convergence and 3, 6, 9, 12 Δ of divergence (Fig 4B). The vergence demand was increased gradually by symmetric opposite shifts of the images on the static stimulation LCD monitors every 1 sec. Each single step was either 2.0 Δ of convergence or 1.5 Δ of divergence). Immediately after reaching the desired relative vergence level, one of three sequences of progressively impaired images was presented to the non-dominant eye (Fig 4C). The progressively degraded images were changed every 1 sec. When the fixation image deterioration exceeded the dichoptic masking threshold, monocular suppression was activated and the fusion was disrupted. In the absence of the disparity signal, disparity vergence was unable to maintain the forced vergent position and there was movement of the non-dominant eye (Fig 4D). When the vertical red line (Fig 5B), initially located in the middle of the violet box (horizontal angular sub tense of 1.5 degree) (Fig 5A) moved out of the box, the subject clicked a button connected to the computer to indicate exact point of fusion loss. In the fixation images there were three boxes with three vertical lines, and only one of them served randomly as an indicator of leaving forced vergent position in the particular measurement. This design helped to maintain the subject’s visual attention to inherently unattractive visual targets. The measurement under each particular condition (relative vergence demand level vs. type of artificial degradation of image) was done only once per subject to minimize the time of visual concentration. The previous version of our methodology was described in [27, 28].

### Participants

Twenty-four threshold measurements were made in each subject. Control software created an automatically random order of measurements by combining 8 levels of vergence demand and 3 modes of the image degradation for each of the 60 experimental subjects. The examiner, as well as the subject, were blind regarding the particular measurement condition. The experimental group consisted of ophthalmologically healthy young adults (17 - 38 years old, median age 21.0 years), 20 males (20 - 33 years old, median age 21.0 years) and 40 females (17 - 38 years old, median age 21.0 years). The exclusion criteria were best corrected visual acuity (BCVA) in worse eye below 0,0 logMAR (ETDRS optotype at 2.0 m, 85 cd/m^2^, VectorVision, Greenville, OH, USA) spherical correction in worse eye above +1,0 D or -3.0 D respectively, cylindrical correction in worse eye above ± 1.0 D, phoric angle above ± 6 Δ, any tropic angle (standard alternate prism cover test at 4.0 m and 0.3 m) and occlusion therapy, strabismus, serious eye disease, any disease of CNS, thyroid or diabetes. All adult experimental subjects, or parents of minor subjects, were fully informed and signed written informed consent prior to the experimental procedure.

### Statistical analysis

The arithmetic mean and standard errors of each group were computed as descriptive statistics. A Shapiro-Wilks test was used to assess normality of the primary data distribution pattern. The relationship between FCT and experimental mode, divergence/convergence and relative vergence demand was analyzed using a repeated measures ANOVA model with subsequent paired t-test for final mutual comparison of the groups. The identification of mutually homogeneous outcomes of experiments was computed using a Tukey post hoc test for paired data. Correlation of image degradation among experimental modes was analyzed using Spearman’s rank correlation coefficient. Statistical analysis was carried out using SPSS 22.0.0.1 (IBM Corporation, 2014) and Statistica 12 (StatSoft Inc., 2013).

## Results

**Figure 6:**
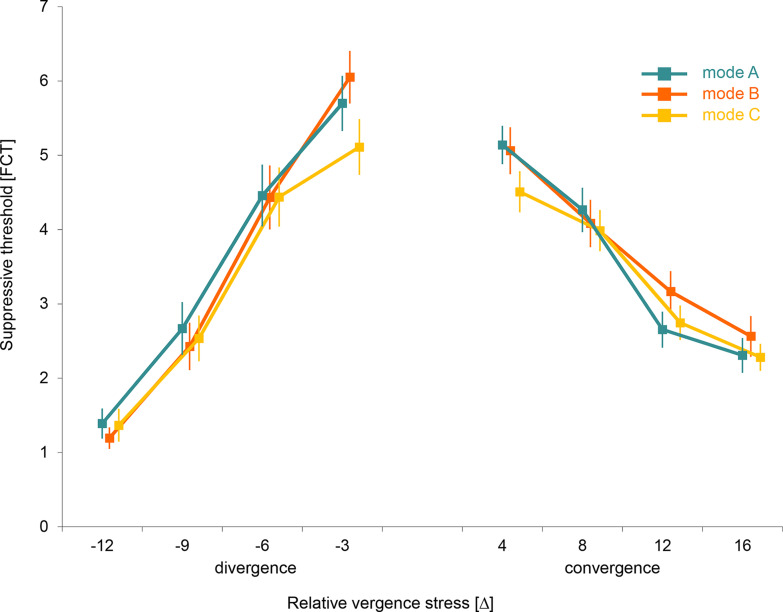
Figure 6. Mean suppressive thresholds in relation to relative vergence demand and degradation mode of non-dominant eye image.Verical bar = standard error of mean.

A consistent decrease of the suppressive thresholds with the increase of vergence demand was found. Some differences in suppressive thresholds were found between the modes of image degradation. The mode C image lowered suppressive threshold more than the mode A or B images at the lowest levels of convergence as well as of divergence demand (Fig 6). 

Table 1 summarizes complex ANOVA model quantifying contribution of experimental treatments to the total experimental variability. The most significant proportion of the total experimental variance was explained by the influence of interaction between direction (divergence and convergence) and vergence stress gradient (91.6%, p < 0.001), followed by the influence of the relative vergence stress (5.9%, p < 0.001) and experimental mode A-B-C (0.9%, p = 0.027). The influence of the other treatment-related interaction items was not statistically significant.

**Table 1:** Repeated measures ANOVA based on full factorial model of the experiment

**Variability source**	**Sum of squares**	**Sum of squares (%)**	**P-value**
Mode A,B,C [1]	25.7	0.9%	**0.027**
Divergence/Convergence [2]	2.4	0.1%	0.761
Relative vergence demand [3]	159.3	5.9%	**<0.001**
[1] * [2]	2.1	0.1%	0.678
[1] * [3]	18.6	0.7%	0.172
[2] * [3]	2487.1	91.6%	**<0.001**
[1] * [2] * [3]	21.2	0.8%	0.106
Total	2716.4	100.0%	

Summary statistics of threshold values over gradient of vergence stress confirmed a highly significant decrease with increasing vergence stress (p < 0.001 in all experimental modes) (Table 2). The decrease (calculated over the whole gradient) was significantly higher in case of divergence than convergence variant (p < 0.001 in all experimental modes). The influence of the relative convergence equals the effect of relative divergence in lower levels of vergence. In contrast, the different impacts were measured in the higher stress levels. Divergence stress decreased the mean suppressive thresholds approximately one and half times more than the equivalent degree of convergence load, irrespective to the experimental degradation mode.

**Table 2:** Mean suppressive thresholds in relation to vergence demand and mode A, B, C of non-dominant eye image degradation (ANOVA models)

		**Suppressive threshold (FCT)**		
**Relative vergence demand**		**MODE A**	**MODE B**	**MODE C**
		Mean (SE)		
**Divergence**	-3 Δ	5.70 (0.37)^A^	5.96 (0.35)^A^	5.00 (0.37)^A^
	-6 Δ	4.46 (0.42)^B^	4.37 (0.42)^B^	4.34 (0.39)^B^
	-9 Δ	2.67 (0.36)^C^	2.40 (0.31)^C^	2.47 (0.30)^C^
	-12 Δ	1.39 (0.20)^D^	1.19 (0.14)^D^	1.32 (0.22)^D^
Statistical significance of suppressive threshold decrease over divergence demand^1^		**F (df: 3, 57) = 47.8 (p < 0.001)**	**F (df: 3, 57) = 63.5 (p < 0.001)**	**F (df: 3, 57) = 31.9 p < 0.001**
**Convergence**	4 Δ	5.14 (0.26)^A^	4.99 (0.31)^A^	4.40 (0.27)^A^
	8 Δ	4.27 (0.30)^B^	4.02 (0.31)^B^	3.89 (0.27)^A^
	12 Δ	2.65 (0.24)^C^	3.12 (0.27)^C^	2.67 (0.23)^B^
	16 Δ	2.31 (0.24)^C^	2.53 (0.27)^D^	2.22 (0.18)^C^
Statistical significance of suppressive threshold decrease over convergence demand^1^		**F (df: 3, 57) = 59.4 (p < 0.001)**	**F (df: 3, 57) = 17.1 (p < 0.001)**	**F (df: 3, 57) = 35.7 p < 0.001**

^1^ p-values of FTC decrease was calculated by r epeated measures ANOVA with a full factorial model.

^A, B, C, D^ the same letter marks mutually homogeneous groups without statistical significance of difference (Tukey post hoc test).

SE, standard error of mean, df: degrees of freedom of the global ANOVA model F test.

Mode A: reduction of both contrast and high spatial frequencies, mode B: reduction of contrast, mode C: reduction of high spatial frequencies. Mode A: reduction of both contrast and high spatial frequencies, mode B: reduction of contrast, mode C: reduction of high spatial frequencies.

The statistical data in Table 3 gives P values of the differences between experimental modes in the blur needed to disrupt fusion, as a function of the level of vergence demand. Mode C was found to be the most sensitive to the vergence demand. In this case, a significant decrease of suppressive thresholds was detected in the lowest levels of the vergence demand, both in divergence (level -3 Δ) and convergence variant (level +4 Δ). At these points, the threshold level of experimental degradation in mode A protected from suppression was significantly different (lower) than outcomes of mode A and B. Modes A and B were not significantly different in any of the tested levels of the vergence demand.

**Table 3:** Statistical significance of differences in the suppressive thresholds of experimental degradation modes with respect to vergence demand levels

		p-value of paired t-test		
Relative vergence demand		**mode A vs. B**	**mode B vs. C**	**mode A vs. C**
**Divergence**	-3 Δ	0.468	0.007	0.030
	-6 Δ	0.774	0.921	0.658
	-9 Δ	0.377	0.804	0.512
	-12 Δ	0.184	0.508	0.715
**Convergence**	4 Δ	0.637	0.048	0.003
	8 Δ	0.370	0.698	0.231
	12 Δ	0.051	0.054	0.921
	16 Δ	0.292	0.203	0.659

**Figure 7:**
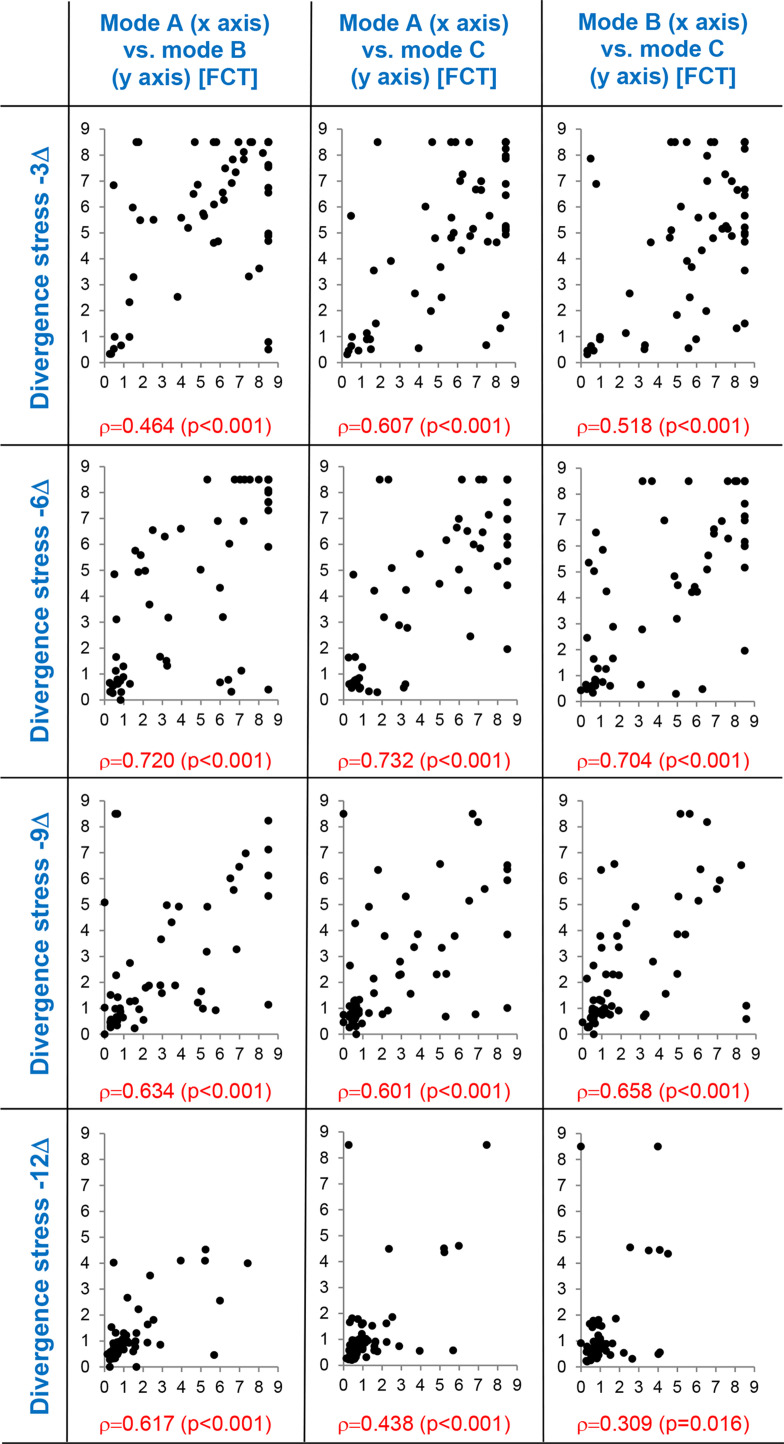
Correlation diagrams of suppressive threshold values between experimental degradation modes A, B, and C during divergence demand.

**Figure 8:**
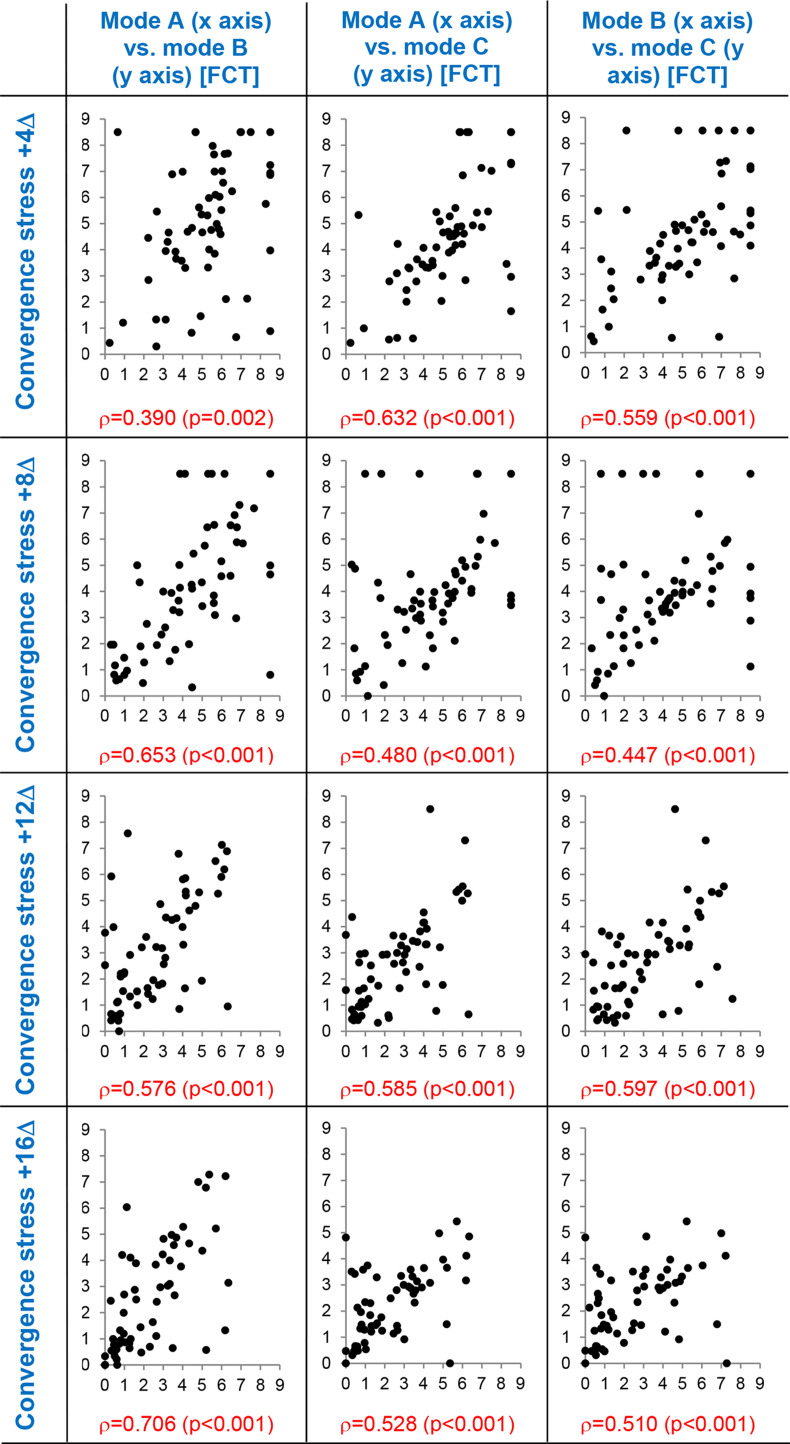
Correlation diagrams of suppressive threshold values between experimental degradation modes A, B, and C during convergence demand.

Supporting correlation diagrams of suppressive threshold values obtained in the three experimental modes A, B, and C are depicted in Figs 7 and 8. Correlation patterns document significant mutual relation of threshold values obtained in the experimental modes which is valid in all levels of the vergence demand gradient. Individual values of the correlated parameters are mutually significantly associated. This result justifies application of paired statistical methods in the evaluation (rmANOVA and paired t-test). The differences among experimental variants are thus assessed on the basis of individually calculated differences of values.

Suppressive thresholds on axes x and y are expressed in fusion cover test (FCT) units, ρ = Spearman’s correlation coefficient (p-value), dots represent the individual data. Mode A: parallel reduction of contrast and high spatial frequencies, mode B: isolated reduction of contrast, mode C: isolated reduction of high spatial frequencies.

Suppressive thresholds on axes x and y expressed in fusion cover test (FCT) units, ρ = Spearman’s correlation coefficient (p-value), dots represent the individual data. Mode A: parallel reduction of contrast and high spatial frequencies, mode B: isolated reduction of contrast, mode C: isolated reduction of high spatial frequencies.

All subjects reported that just before disruption of fusion, the red vertical line (Fig 5B) moved very slowly and/or with a fluctuating horizontal drift toward the violet reference box’s margin (Fig 5A). The prevailing direction of the drift was opposite to the direction of vergence demand. 

Because the effect of the mode is most important in this study, this effect can be specified in terms of the “effect size”, i.e. difference between modes / pooled standard deviation. Effect size of mode within divergence and convergence demand is given in Table 4 below.

**Table 4:** Effect size of mode within divergence and convergence demand

**Relative vergence demand (experimental treatment) **	**Image degradation (FTC): comparison of outcomes of experimental modes A–B–C**		
	**MODE A**	**MODE B**	**MODE C**
FTC decrease (standardized) over divergence demand gradient (-3 → -12)	-1.87	-2.06	-1.59
FTC decrease (standardized) over convergence demand gradient (4 → 12)	-1.08	-0.80	-0.75

## Discussion

In the current study, the combination of vergence and differential blur demanded fusion in ophthalmologically healthy young volunteers. The endpoint of the stepwise progression of this demand was the disruption of fusion. The artificial manipulation of the image quality of the non-dominant eye while the dominant eye was continuously stimulated by the normal image resulted in a progressive increase in the interocular blur difference. Exceeding the suppressive threshold level of differential blur in combination with vergence demand disrupted binocular fusion. Presumably, the degraded monocular signal is suppressed in the population of monocular V1 neurons before binocular summation [29], prior to disparity detection. The absence of a disparity signal caused the disruption of disparity vergence mechanisms and consequently the suppressed non-dominant eye left its forced vergent position. 

Clinical practice offers examples of exclusive visibility of monocular images when interocular blur difference seriously handicaps the signal from the other eye. A common example is the percept during the alternate cover test routinely performed in strabismological practise. Form-deprived and heavily blurred signal from the covered eye conforms the definition differential occlusion [30] and generates transient interchanging monocular suppressions. The presence of suppression was confirmed by experiment when sharp texture on occluder corresponded with occluded texture instigated binocular rivalry [31]. Similarly, persistent monocular suppression is seen in monovision [32], i.e., contact lenses modality wearing strategy with one eye corrected appropriately to distance and the second eye covered by lens with presbyopic addition. These phenomenon are analogous to IOBS documented in current research.

### Impact of contrast edge’s spatial frequency bandwidth on fusional stability

The degraded images presented to the non-dominant eye in the current research are dichoptically masked. Mode A was created with the intention to simulate a natural blur generated by uncorrected refractive error. Pseudo naturalistic degradation of mode A proved its consistent and robust suppressive potential in our experiments. Similar suppressive potential is related to the interocular blur difference in anisometropia [20]. Consistency and robustness of this potential is widely confirmed by clinical evidence [33]. Regardless of the complex nature of amblyopopia [34, 35], it seems highly plausible IOBS is a considerable contributing factor in the pathological effect of anisometropia [16]. 

The pixel luminance pattern of our experimental images differs amongst three degradation modes. Changes of retinal image generated by mode A are characterized by a balanced combination of contrast and high-spatial frequencies decay [36]. Such an artificial deterioration of a natural scene’s image is partially analogous to the luminance modulated mask with low-pass filtering of high-spatial frequencies used in laboratory visual experiments. Mode B, artificially modeling blur with isolated progression of target’s contrast reduction resemble a luminance modulated mask. Finally, mode C, is artificially modelling blur with isolated reduction of high-spatial frequencies achieved by low-pass filtering with progressive lowering of upper frequency limit. Because the contrast (i.e. luminance modulation of the blur signal) in modes A and B is reduced homogenously over whole images, the participation of the contrast-modulation paradigm requiring second order processing is improbable.It is, therefore, plausible, that the IOBS in current experiments is processed by neuronal interactions of bandpass selective neuronal populations (spatial frequency tuned channels in computational terminology) [37] in early binocular rivalry levels. The key role of inhibitory neuronal interactions at primary visual cortex before binocular summation in discussed masking phenomena was originally proposed by Breitmeyer and Ganz [38] and consequently confirmed by some others [39, 40]. 

During low vergence demand in our experiments, the significant suppressive threshold differences between mode C and mode B were demonstrated. Intentional exclusion of an image’s details from the monocular signal in mode C by low-pass filtering inactivated high-spatial frequencies tuned channels. It was associated with the increase of suppressive potential when compared to homogenous lowering of luminance contrast over all spatial frequency tuned channels in mode B (see Fig 6). The images in mode A still contain some high-spatial frequency information, albeit degraded in contrast (compare right panels in Fig 2) and had, like those of mode B, lower masking potency than the purely low-pass filtered images with full contrast in mode C. The low-pass filtering effect affected mode A and mode C differently. The disappearing of high-spatial frequencies in mode A is less progressive than in mode C because of adjunct effect of luminance contrast reduction in mode A.

In other words, the activity of high-spatial frequency tuned channels, even if stimulated by a signal with lowered luminance contrast, preserved a degraded monocular image from IOBS more than activity of low-spatial frequency tuned channels sufficiently loaded with high contrast stimuli. An analogous protective effect of higher - spatial frequencies to stereo acuity demanded by low luminance contrast was described by Schor and Heckman [36] and confirmed by Reynaud and Hess [35] . This indicates that low - spatial frequencies carrying coarse visual information and high-spatial frequencies carrying fine details have different functions in visual processing. Our observations are in agreement with the feedback model of visual analysis first proposed by Hughes and colleagues [41] and reviewed lately by Bar [42]. This theory of sequential cortical processing of visual information was inspired by the pioneering work of Navon in precedence of an image’s global structures analysis [43]. Ample psychophysical evidence collected since has suggested low spatial frequencies, preferably on the peripheral retina, are conveyed through the magnocellular pathway rapidly to higher-order areas in parietal cortex for computation of the initial coarse parsing of the visual scene. Consequently, results from first-pass processing are re-injected in top-down feed-back and modulate fine image analysis of high-spatial frequency signal available in parvocellular structures of V1 [5, 44-46]. This fundamental division of spatial frequency processing enables the differences in their impact on fusional mechanisms. 

### Influence of vergence demand on fusional stability

Artificial low-pass filtering detracts high-spatial frequency information and violates perceptual (incl. fusional) prominence of unaffected stimuli conforming to the spatial frequency statistics of natural scenes [17]. Based on this assumption, in our experiment we could speculate about the nature of fusional deterioration related to the progression of vergence demand. The impact of isolated reduction of high-spatial frequencies was measurable only in low vergence demand levels. During higher levels of vergence demand, the fusional stability was much more seriously affected, irrespective of the type of blurring mode. Considering Collewijn et al.’s hypothesis proposing that vergence demand enlarges Panum’s areas and thus revealing their true potential size [47], we could anticipate simultaneous expansion of retinal receptive fields. The consequent decline in the retina’s ability to capture the high-spatial frequency signal, amplifies the suppressive potency of the artificial blur of stimuli. Consequently, distortion of spatial frequency statistics makes the image more prone to its suppression than to its changes when generated solely by artificial low-pass filtering. 

In accordance with previous assumptions, there are other studies matching the vergence demand progression with deterioration of stereoscopic vison quality. Initially, Blakemore [48] and Abd-Manan [49] proved that stereoacuity decreases with increasing fixation disparity related to forced vergence demand. Different impact of convergence and divergence demand on local and global stereopsis (real world Frisby test vs. random-dot tests) have been described previously [50, 51].

There is a paucity of experimental data regarding the impact of increasing vergence demand on binocular suppressive mechanisms (incl. IOBS). Nevertheless, suppression accentuated by vergence maladaptation is a key mechanism underlying clinically important pathological processes, like decompensation of phoria. Our experimental results parallel clinical experiences, where the decrease of the visual system resistence to monocular suppression is tightly related to increasing vergent demand. Forced divergence has a more pronounced impact on binocular fusion stability than convergence does. This is consistent with generally accepted clinical experiences. Esophorias more readily decompensated to manifest convergent strabismus because of the smaller reserve of relative divergence compared with convergence reserves. 

### Suprathreshold fluctuation of indicator line inside the reference box

During the short measurement period immediatelly before disruption of disparity vergence mechanism, subjects invariably saw very slow and/or fluctuating horizontal drift of the red indicator line toward the reference box’s margin. The sensorial fusion was still maintained in this phase because of the presence of relative disparity vergence. The drift of the indicator line was markedly slow and with direction reversals inside the reference box. Immediately after suppressive threshold exceeding, the fluctuating and/or slow drift was replaced by vigorous directed movement outside the box caused by the unlocking of the forced vergence position of the suppressed eye. The horizontal dimension of the reference box was larger (1.5 deg) than the conventionally accepted dimensions of Panum’s areas (a few tens of minutes of arc). An analogous observation was published by Hyson, Julesz and Fender [52]. They directed eyes of probands to a forced vergent position during tests with random-dot stereograms. When the visual axes were misaligned by up to 2 degrees, the global stereopsis was still maintained. They concluded that the retinal correspondence, underlying binocular stereopsis, can shift in normal observers [52]. An alternative explanation argues that these experiments reveal the enlarged true potential size of Panum’s areas [47].

## Conclusions

The study confirmed our hypothesis. Different modes of artificial interocular blur imbalances in an image’s quality does differ in their capacity to disrupt binocular fusion. The conclusion is based on the measurements of two dimensional affection of fusional stability accentuating the prominent influence of the high-spatial frequency content of a natural scene’s image to fusional stability. Both, artificial blur generated by low-pass filtering, and plausible enlargement of Panum’s areas induced by forced relative vergence, violated binocular fusion by a paucity of pictorial details. Such a statement well-mirrors widely confirmed clinical evidence of antisuppresive and fusion supportive potential of the best spectacle correction on reinforcing an image’s details and relative vergence training reducing vergence stress. Our results, emphasizing the antisuppressive potency of targeted stimulation of high-spatial frequency tuned channels, could lead to optimization of treatment strategies including sensory processing rehabilitation and may improve the outcome of modern pleoptics.

### Ethics and Conflict of Interest

The authors declare that the contents of the article are in agreement with the ethics described in http://biblio.unibe.ch/portale/elibrary/BOP/jemr/ethics.html and that there is no conflict of interest regarding the publication of this paper.

### Acknowledgements

The technical part of the research was supported in part by a grant from the Czech Science Foundation No. GA17-05840S Multicriteria optimization of shift-variant imaging system models. 

We are grateful to Stuart J. Judge, M.A., Ph.D. (neurosci.) (Emeritus Reader in Physiology, Department of Physiology, Anatomy and Genetics, University of Oxford, Oxford, UK) and Mr. Richard Harrad M.A.(Cantab.), M.B., B.S.(Lond.), M.R.C.P., F.R.C.S., F.R.C.Ophth (Honorary Reader, Bristol Medical School, University of Bristol, UK) for their kind comments and advices. We thank Katerina Mala and Marcela Dostalkova for their valuable help with data collection.
